# The Relation of the Autonomic Nervous System to Clinical Medicine

**Published:** 1926

**Authors:** J. R. Charles

**Affiliations:** Physician to the Bristol Royal Infirmary


### The Bristol Medico-Chirurgical Journal

"*Scire est nescire, nisi id me*
*Scire alius sciret.*"

AUTUMN, 1926.

### THE RELATION OF THE AUTONOMIC NERVOUS SYSTEM TO CLINICAL MEDICINE.

Presidential Address, delivered on June 30th, 1926, to the Bath and Bristol Branch of the British Medical Association.

BY

J. R. Charles, M.A., M.D. (Cantab.), F.R.C.P.,
*Physician to the Bristol Royal Infirmary.*

---

The autonomic nervous system plays such a paramount part in the manifestations of disease processes in the body, and the recognition of this is so important, that I have ventured to choose this subject for my address. The problems which it embraces are, however, so vast, and in many cases so little understood, that it will be only possible to touch the fringe of the subject; especially is this so when we realise what the autonomic nervous system does.

It influences those functions of the body without which the life of man cannot exist. It controls the metabolism of the body. It directs the action of all glands, whether these have an external or internal secretion. The action of the heart, vascular system, and musculature of all the internal viscera are under its sway. Directly, or indirectly, it affects the mind. It is powerfully influenced by the mind. The mind, acting through it, influences the metabolism of the body. Through it the individuality of man is largely expressed. Through it we are born, and through it may die.

It is the primitive nervous system, much older in evolutionary development than the rest of the nervous system, to which so much attention has been given. We know much about the degenerations of the skeletal nervous system. What do we know about neuritis and localised degenerations of the peripheral parts of the autonomic nervous system ? Yet we have no reason for supposing that such lesions may not occur just as they do in the somatic nervous system.

Again, how much there is to be studied in the reflexes of the autonomic nervous system. These, or actions similar thereto, are very numerous, and probably as time goes on their clinical application will be more and more utilised. Concentration of thought on the working of these systems tends to make one realise how disease processes affect the body as a whole, and so directs our attention more and more to the patient who is suffering from the disease, rather than the disease merely considered as an entity in itself.

The autonomic nervous system is divided into three main groups : the cranial (mainly vagal), the thoracico-lumbar, and the sacral.

The vagal is anabolic, protective, and a builder of reserve.

The thoracico-lumbar, or sympathetic, is katabolic and largely useful in self-protection, for through its action primitive man could defend himself by fight or flight, since it directly or indirectly increases skeletal muscular force, increases the heart rate, dilates the bronchi, allows more blood to go to the muscles, contracts the vessels of the skin, promotes perspiration, and restrains the activity of the gastro-intestinal functions. At the same time, it liberates more food in the form of sugar for the energy of the muscles. It therefore acts as a preserver of the individual.

Phylogenetically the vagal system is the older, the sympathetic system developing later, a point which has a bearing on the innervation of the sweat glands.

The sacral overflow is largely concerned in the continuance of the race, and is therefore the preserver of the race. In almost every form of disease the symptoms are largely of vegetative nervous origin. Through this system disease manifests itself.

The parasympathetic and sympathetic divisions are almost entirely antagonistic, and it is not surprising that with over-action or under-action of either part in disease we meet with the most diverse combination of symptoms.

Of the anatomy of the system we know much, but how little of the pathology or morbid anatomy. How often at autopsies is any attention whatever devoted to it ? Here lies a great future, the unravelling of a vast and difficult problem. We realise how little we know, and we grope in the dark, feeling that if only we knew how to control, to stimulate, or inhibit at will the different parts of the diverse sections of this mighty system then, as physicians, we could do what we now only dream of. In whichever direction we turn in disease we see the system profoundly affected, and we realise how remote or how widely spread the resultant manifestations of primarily localised disease may be.

DR. J. R. CHARLES

There are many observers who consider that states of over-balance of the vagal or sympathetic systems exist among otherwise normal people. To these states the terms Vagotonia and Sympatheticotonia are applied.

Atropin paralyses the terminations of the parasympathetic, pilocarpin stimulates the parasympathetic. Eppinger and Hess found a group of people who were relatively insensitive to the effects of atropin, while unduly sensitive to pilocarpin. These they classified as cases of Vagotonia.

A difficulty, however, in accepting this view in its entirety is the action of pilocarpin on the sweat glands, which are supplied by the sympathetic nerves. The case for Vagotonia appears to have been overstated; but although this is so, there appear to be clinical conditions, at any rate, in which the vagal influence is over-active; *e.g.*, many cases of asthma, which are relieved by diminishing the irritability of the vagus by atropin, or by stimulating the antagonistic sympathetic by adrenalin, or by giving calcium, are almost certainly examples of a vagotonic syndrome. Probably anaphylaxis and serum disease are also parasympathetic syndromes, and are relieved by the same remedies.

It is perhaps noteworthy that conditions of Sympatheticotonia or Vagotonia can be observed in animals. This may involve differences even in the mode of death. For it is curious that a cat should die by a different mechanism from chloroform poisoning than does a dog from the same cause. A cat is sympatheticotonic, and probably dies by ventricular fibrillation due to sympathetic stimulation, while a dog, which is vagotonic, dies from vagal inhibition.

Observations such as these lead us on to consider for a moment some of the manifestations of hyper-irritability of the sympathetic system in man.

Among the main manifestations we have a whole series of events which are essentially katabolic in nature, viz. prominence of eyeballs, dilatation of pupils, diminished secretions of lachrymal, salivary, nasal, gastro-intestinal tracts, the latter leading to hypochlorhydria and retarded digestion, diminished peristalsis of stomach and intestines, leading to one form of constipation, rapid pulse, and sometimes elevation of blood pressure, increase of blood sugar, contraction of uterus, increase of temperature (due to increased chemical changes) and vaso-constriction of cutaneous vessels, goose flesh, or increased sweating, and increased adrenal and thyroidal secretions. It is clear that these are outstanding features in many clinical conditions, and are also observable in very variable degree in people who are not technically ill, but who perhaps never seem quite fit. Such people appear to be much more susceptible to the effects of various viscerogenic reflexes than others.

These reflexes are extremely numerous. Reflexes occur between the sympathetic and the spinal nerves, not only in the segment of the cord which receives the afferent impulse from the viscus concerned, but also in segments far removed, the impulse passing by intercalated neurons in the cord. The same applies to the parasympathetic system. Thus we see that pressure in the nose may, by irritating the 5th nerve, produce asthma via the vagus.

Every important viscus is able to produce reflexes through afferent sympathetic and efferent spinal nerves in definite skeletal structures. If the viscus be acutely inflamed these reflexes produce alteration in sensation ; if chronically inflamed, trophic changes.

There are three main types of reflexes: viscero-motor, viscero-sensory, viscero-trophic.

*The viscero-motor* are represented by spasms in the skeletal muscles in the corresponding segments of the inflamed viscus. They are seen in the rigid abdomen, rigid thorax, in acute lung and abdominal diseases, spasm of the lumbar muscles in some renal complaints, and so on. It is characteristic of these that the muscle as a whole does not contract, but mainly that part which is supplied from the corresponding segment. The sterno-mastoids and trapezii may be similarly affected in acute cases of phthisis. If the condition persists it leads to a viscero-trophic effect, with wasting of the muscles.

*The viscero-sensory reflexes* are seen in the painful areas described by Head. Pain, however, from a diseased viscus may radiate over a much wider area than that supplied by the corresponding segmental skin area, as is seen in angina pectoris. With a relatively slight stimulus the pain may be precordial only, or with increasing stimuli, over the inner side of the left arm, or right down the inner side of the arm to finger-tips, or with even a greater stimulus down the right arm also. Moreover, similar reflex pains may be felt in later life, when there is then no longer any activity in the disease of the viscus. Such pains may occur if the patient is from any cause below par, or with changes in the weather, or when fatigued. The reasons for these pains frequently baffle us. The explanation of this probably is that when the pathway for pain has been opened by an adequate stimulus, the threshold of response to stimuli which ordinarily would produce no pain may be permanently lowered, so that pain results from a stimulus owing to a hyper-sensitiveness induced in the nerves concerned.

*The viscero-trophic reflexes.*—These are best seen in chronic visceral disease, particularly chronic pulmonary tuberculosis. Here we have the wasting of the muscles either in front or behind the upper part of the chest in apical phthisis, and the hilus "saucer of degeneration" described by Pottenger in the inter-scapular space in cases of hilus phthisis. In chronic disease of the kidneys, *e.g.* tuberculosis, there may be definite muscular wasting of the lumbar muscles. We see, therefore, that such a reflex may produce structural loss of tissue.

Perhaps one of the most striking features of clinical medicine is the variability of symptoms produced by the same disease in different people, a feature which adds so much difficulty to accurate diagnosis. This variability appears to be largely due to the difference in susceptibility of the "autonomic nervous system." Examples are common enough. Take, for example, two cases of tuberculous peritonitis with the same pathological changes. One may have no symptoms to speak of, the other may suffer from such acute symptoms that the surgeon regards the case as an "acute abdomen," or the gastric ulcer which gives rise to no symptoms till it ruptures, or the gangrenous appendix which behaves in a similarly erratic manner, the latent pleurisy with effusion, or the terminal pneumonia unassociated with cough, expectoration, elevation of temperature, or increased respiratory rate.

It is true, of course, that other factors must be taken into consideration, such as the activity of the endocrine glands, and also the colloids of the cells of the body and their ionic content. Indeed, there seems to be some reason for believing that this latter condition may not only be responsible for a damping off of symptoms, but even for a reversal of action, *i.e.* an effect from a given stimulation just the opposite from that which might have been expected.

Zondec found that stimulation of the sympathetic going to the heart throws out calcium into the perfusate, which if perfused through another heart gives the same result as sympathetic stimulation. Further, that if the vagus is stimulated potassium is thrown out into the perfusate, which if perfused through another heart gives the same result as if the vagus had been stimulated. Kolm and Pick showed that a hypersensitisation of one component of the autonomic nervous system such as might be produced by excessive calcium or potassium ionic content of cells may bring about a reversal of action, *i.e.* a parasympathetic effect when a sympathetic effect would have been expected, or vice versa.

Perhaps this is one of the reasons which may explain the abnormal idiosyncrasy which some patients exhibit towards certain drugs. I recently happened to be with a patient who was going to have a tooth removed under a general anæsthetic. She was given some atropin beforehand, and very soon afterwards she complained of cramp in all her limbs, which complaint continued until she was under the anæsthetic. On recovering from this she was in a condition closely resembling severe tetany, with such marked carpo-pedal spasms that it was with the greatest difficulty the fingers and feet could be straightened out. This condition continued for an hour or so. I was convinced that this state had been produced by atropin. No other drug had been given. How could such a clinical effect be produced ?

Tetany is a condition which is supposed to be due to parathyroid and calcium deficiency with consequent hyper-irritability of certain parts of the nervous system leading to the well-known spastic symptoms. Possibly these may prove to be due to hyper-irritability of the sympathetic muscular nerves, the nerves of plastic tone. Similar spasms have been seen as the result of ergotism, and ergot stimulates the sympathetic. Atropin paralyses the parasympathetics, and there is no evidence of any parasympathetic innervation of the skeletal muscles. I can only, therefore, assume that in this case there was a reversal of action.

The episode to which I refer occurred about four months ago. The patient got well. A few days ago she had a most tempestuous voyage at sea with constant vomiting. She wrote to me saying that when she was so sick she lay on deck quite unable to help herself because "the cramps" had returned. This event supports, I think, the view that the condition was a true tetany.

T. P. Martin [1] has recently adduced the view that tetany is myogenous in character and not neurogenous. But there are other phenomena associated with tetany which appear inexplicable along the line of myogenic origin. I refer to changes in the skin, excessive sweating, changes in the hair and nails, and dysmenorrhœa. Moreover, the paroxysms may be excited by emotion. These are symptoms attributable to sympathetic over-action. Perhaps another explanation is that the atropin produced a sudden parathyroid deficiency. Both physical and psychical stimuli vary in their effects, some showing a preference for stimulation of the parasympathetic nerves, others of the sympathetic.

When we consider for a moment some of the symptoms of common diseases, we notice what a difference there is in the manifestations of toxæmias.

Compare two ordinary diseases, let us say typhoid and scarlet fever. Typhoid affects the alimentary canal, where the parasympathetic nerves are potent, and we meet with dry mouth, mental depression, sluggish eyes, and a pulse rate characteristically slow in proportion to the temperature—all suggestive of vagal predominance. Indeed, one of the diagnostic tests for typhoid is based on this. Marris's test consists of injecting 1/33rd gr. of atropin subcutaneously, under certain conditions. In a normal person the pulse rate would be raised markedly, say 30 beats per minute, owing to its antagonising action on the vagus. In typhoid, however, the trouble in the intestine appears to exert so marked a stimulation of the vagal endings in the intestine that the same dose of atropin fails to inhibit it in the same degree, and a pulse rise, therefore, of under 15 per minute, in a suspicious case, makes the inference that the disease is one of the typhoidal group very strong. This is therefore a test for abnormal vagus stimulation rather than a specific test for typhoid.

How frequently also is mental depression associated with disease of the intestine and liver. We have only to think of those people who suffer from membranous colitis. Diseases of the liver are notoriously depressing, the word "melancholia" itself being derived from the bile; and circulating bile salts produce also the characteristic slowness of pulse.

Now in scarlet fever we see an entirely different clinical picture—bright eyes, red skin, increased respiration, and a pulse rapid in proportion to the temperature—a disease in which there appears to be over-stimulation of the sympathetic; in fact, its manifestations are very similar in many ways to belladonna poisoning.

One wonders whether those toxæmias which are associated with a sympathetic over-stimulation may be those diseases in which nephritis is more prone to arise. The sympathetics are said to furnish both vaso-dilator, as well as the vaso-constrictor fibres to the kidney, and a definite vaso-dilatation would involve these organs being subjected to a greater amount of circulating toxin.

Bayliss found that stimulation of the posterior spinal roots either before or after passage through the ganglion produced dilatation of the vessels in the area supplied by the sensory nerve. These impulses he called "antidromic," or reversed impulses. Thus the same nerve fibre functions in opposite directions at the same time, sending sensory impulses upwards, and vaso-dilator impulses downwards. The eruption of herpes zoster is explained in this way, and it is interesting to remember that an association between herpes zoster and varicella has frequently been observed.

### Heredity.

Many of the diseases which are transmissible by heredity are diseases largely of the autonomic nervous system, as, for instance, some forms of diabetes, gout, migraine, and asthma. To these perhaps may be added the myopathies. A dual innervation of the skeletal muscles has been fairly conclusively proved by Hunter, who drew the deduction that the skeletal muscles of vertebrates consist of two sets of muscle fibres, with different innervation and different functions. One of these nerve supplies is from the anterior horns and produces "contractile" tone, the other, being sympathetic in origin, producing "plastic" tone, *i.e.* a tone which maintains any posture when it has been assumed—a fixation tone.

Edwin Bramwell in the Bradshaw Lecture (Nov. 5th, 1925) brings forward very strong reasons in support of the view that the muscular dystrophies may be due to an affection of the sympathetic nervous system. The muscles primarily involved are those which are developed early in the fœtus, those which no longer fulfil the same important functions which they did in the earlier stages of evolution. Take, for example, the extrinsic muscles of the shoulder girdle, the serratus magnus, latissimus dorsi, rhomboids, and lower part of the pectoralis major. These, instead of forming a slinging system, as in a four-footed animal, now act mainly as fixing muscles for the arm, and also show atrophic changes early in the disease.

Atrophy and hypertrophy of the bones, a marbled condition of the skin, indicate that the disease is more than a localised muscular dystrophy. If, then, it is possible for the skeletal muscular sympathetic fibres to be susceptible to an early death, it is not surprising to find that an infant may be born with a preponderance of one part of the autonomic nervous system over another, or in other words that it may be vagotonic or sympatheticotonic.

Many of our emotions are closely associated with instinctive acts which have come down to us from remote ancestry, and are analogous to the anatomical structures of our bodies derived by evolutionary changes from lower forms of life and function. These changes act very largely through the autonomic nervous system, the result of many being well recognised, *e.g.* the lump in the throat felt in emotion, the dryness of the mouth through fear, the tongue cleaving to the roof of the mouth, or conversely the thought of something palatable making the mouth water, the soundings of the bowels, the diarrhœa, polyuria of emotion, and so on.

Many of the normal physiological processes may be profoundly modified or disturbed by emotional disturbances. Thus digestion may be completely upset. Not only fear, but anger, has been shown experimentally in animals and in man, with gastric fistulæ, to inhibit gastric secretion, sometimes entirely. Indeed, these emotions may be so disturbing that taking appetising food even some hours later may fail to cause the normal flow of digestive juice ; in other words, the effect of emotion may long outlast the exciting cause. It has been shown also that the flow of the pancreatic juice and of the bile are similarly influenced. During very great excitement gastric and intestinal peristalsis may entirely cease, a fact which has frequently been observed when examining animals with X-rays.

Hurst reports an interesting case of a nervous man whose gastric peristalsis was being examined by X-rays. The operator tapped the man with the screen under his chin, producing fear. All peristalsis immediately ceased, and moreover the stomach was seen to drop, so that he surmises, probably correctly, that the phrase "My heart dropped into my boots" should rather be, "My stomach dropped."

The psychical element must be taken into consideration when abdominal radiograms and gastric test meals are interpreted. Cannon, for example, refers to a woman with digestive disturbances who was sent for special examination by these methods. Her husband got drunk, and she spent a sleepless night before the examination. On examination the following morning no free HCl was found, no digestion of the test meal had taken place, but remains of the supper of the previous evening remained. A repetition of the examination a day later, when the patient was calm and rested, showed normal gastric juice, normal digestion of test meal, and normal peristalsis.

Pain also will stop gastric peristalsis, or may produce nausea or vomiting, hence the expression "a sickening pain." The inhibition effects outlast the cause, a possible explanation of this being that the emotional cause stimulates the adrenals, liberating more adrenin into the veins, which substance, although it produces the same effects as sympathetic stimulation, acts directly on the viscera involved, and not through the nervous system.

By a variety of experimental methods Elliott and others have proved that artificial stimulation of the sympathetic nerves going to the adrenals causes a discharge of more adrenin into the circulation. The intestinal muscle of the rabbit is remarkably sensitive to the effects of adrenin, the normal rhythmical movements being inhibited by a dilution of 1 in 200 millions. Cannon has shown that blood taken from the inferior vena cava of an animal, where the veins from the supra-renal enter it, inhibits the rhythmical movements in the rabbit's intestine, if the animal has been subjected to excitement by etherisation, or fear, or pain, while this is not the case if the animal has not been subjected to these influences, but has merely been passive.

It has further been shown that the emotions produced by fear, rage, pain and also by mental effort produce an increase of sugar in the blood, and very frequently a temporary glycosuria. Four out of nine medical students after a difficult examination had sugar in the urine, while only one of the nine had it after an easier examination (Cannon). Twelve cats (which are markedly sympatheticotonic) when artificially excited all showed marked glycosuria, though they had previously been free from this (Cannon).

One of the functions of adrenin is to liberate sugar from the liver, and sugar is the main source of muscular energy. Therefore perhaps we have one reason for the marked muscular weakness which is associated with Addison's disease, though perhaps it may be shown that other factors are operative, *e.g.* loss of plastic tone from loss of the sympathetic nerve force supplied to the small fibres of the skeletal muscles (Hunter). The low blood pressure would also have a deleterious effect in permitting fatigue metabolites to accumulate. Adrenin itself, moreover, lowers the fatigue threshold of neuro-muscular activity and causes forcible contractions.

Adrenin given in small doses increases the coagulability of the blood, and this may also be produced by splanchnic stimulation, which acts by increasing the amount of adrenin. Probably there is some co-operation between the adrenin and the liver, as this diminution of the coagulation time of the blood does not occur in the absence of the liver. Rage and pain also increase the coagulability of the blood, which is almost certainly due to adrenal discharge acting through splanchnic stimulation.

It is interesting to note that the optic thalamus is the centre representative of the protopathic aspect of peripheral sensibility, as well as the central base of emotive reactions, that Rivers regarded the emotive instincts as protopathic in character, and also that antidromic impulses pass along the protopathic nerves.

The emotional states which are manifested in the sympathetic division of the autonomic nervous system are more intense than those manifested in the other divisions, because the sympathetic is the preserver of the individual. We see an example of this in bad spasmodic asthma. A patient who may be breathing with the greatest difficulty will get complete relief if he receive a severe stimulus of fear. He will therefore run with ease from his burning bedroom, the sympathetic response called into play by the emotion completely overcoming his previous vagotonia.

### The Brain.

Some reference must be made to disorders of the brain and the autonomic nervous system. These disorders are seen both in organic and functional or psychical troubles.

There is almost always some evidence of disturbance of the sympathetic nervous system in hemiplegia, from whatever cause this arises. These phenomena may be increased blood pressure in the affected side, as compared with the other, increased elevation of temperature, and diminished sweating, passing frequently into exactly the opposite condition, viz. diminished temperature, lowered blood pressure, and increased sweating. The early hyperthermal syndrome is attributed to paresis of the sympathetic system and is associated with flaccid paresis, the second stage being hypothermal, analogous to the spastic stage of hemiplegia.

Passing on to psychical considerations, it seems quite possible that dreams, more particularly those which are distressing and associated with states of fear, like night terrors in children, working through the emotional centres, uncontrolled as these are during sleep by the higher reasoning faculties, may lead to definite functional changes in the autonomic system, and perhaps play a part in the ætiology of many obscure conditions; for we have seen that the effect of an emotion outlasts its existence.

Eustace Smith described, in association with night terrors, a condition which he called "mucous disease," in which the patient is languid, irritable, sallow, constipated, with an excess of mucus in the motions, with abdominal discomfort, cyclic albuminuria and vaso-motor disturbances.

The emotions are, of course, phylogenetically much older than the reasoning faculties of mankind. Hence we should expect emotional responses to be more manifest in childhood before the reasoning faculties are developed. The fact that in childhood there is very marked instability of the autonomic system is seen in the liability in the early months of life to spastic conditions of the spincters of the gut, viz. hypertrophic stenosis of the pylorus, of the ileo-cæcal sphincter, and of the pelvi-rectal sphincter, leading to Hirschsprung's disease, whilst later in infancy there is a liability to an irregular form of peristalsis resulting in intussusception.

Even if we consider individuals in health, we meet an infinite number of variations, but certain types stand out. The impulsive, energetic, intense lady who speaks to us quickly and emphatically, though not perhaps very logically, with rapid movements and a frequent slight retraction of the upper eyelids, is she not a physiological example of an inherently over-active sympathetic nervous system? All out-put, katabolic, liable to nervous breakdowns? She may have a feeling of exaltation and well-being, but she is living dangerously, and exhaustion lies in wait, which may lead to disorder of the digestion, or such diseases as diabetes.

As we have seen, the autonomic nervous system is outside the action of the will. But even to this there are exceptions. A case has been recorded by Timme [2] in which a man could at will dilate his pupils, produce goose skin in any part of his body he desired, and raise the hair on his arms. This he did, not by direct voluntary effort, but by intensely imagining the appropriate stimuli which would call forth such reactions. For example, to produce goose skin he imagined his arm thrust into ice-cold water. So we see that imagination may produce effects the nature of which may readily be mistaken from the clinical aspect.

A somewhat interesting point here arises in connection with acting, *i.e.* whether a physical simulation of an emotion tends actually to produce that emotion. Do actors really feel their parts or not ? The question was put some years ago to the leading actors of the day, and the replies were in flat contradiction to one another. Much probably depends on the nervous make-up of the actor, some good actors not only being able to feel their part, but live in it for the time. If so, one would ask, Can these simulated emotions produce the same visceral effects as emotions produced in the usual ways ? Edmund Burke in a passage "On the Sublime and Beautiful" says: "I have often observed that on mimicking the looks and gestures of angry, or placid, or frightened, or daring men I have involuntarily found my mind turned to that passion whose appearance I endeavoured to imitate; nay, I am convinced it is hard to avoid it, though one strives to separate the passion from its correspondent gestures."

William James gives the following explanation of the discrepancy in the replies of the actors: "The visceral and organic part of the expression can be suppressed in some men, but not in others, and on this it must be that the chief part of the felt emotion depends. Those actors who feel the emotion are probably unable, those who are inwardly cold are probably able, to effect the dissociation in a complete way."

Furthermore, there is a disease in which people by continual concentration of their thoughts on their internal viscera bring into the realm of consciousness sensations of movements of these viscera, which normally do not pass the threshold of consciousness. It is possible that normally such impressions do reach the brain, and though unperceived as such, may colour the individuality, probably forming an integral part in the causation of feelings such as "well being," "melancholia," and so forth. Patients whom we call hypochondriacs can, however, give us accounts of sensations from the viscera with an intimate knowledge, of which the normal person knows nothing. They appear to have developed a new sense, which unfortunately increases their discomfort.

There appears to be very strong evidence to support the view that damage to, or irritation of, the different sections of the autonomic nervous system may lead to very definite structural changes. Consider, for instance, the following examples in connection with the vascular system. Lapinski showed that severance of the cervical sympathetic in rabbits led to changes in the arteries which consists of atrophy of the media and thickening of the intima, though in some cases the media was thickened.

In dogs it has been shown by experiments by Manouélian that if a nerve filament be removed which goes to a certain part of the abdominal aorta atheromatous plaques are formed at that spot. Similarly he removed a nerve filament going to the part of the pulmonary artery in a dog. After 65 days a sclerosed patch was found at the part, the pathological changes being similar to those found in human beings in arterio-sclerosis.

Wingate Todd (1913) found arterio-sclerotic changes in a radial artery presumably due to nerve irritation from a cervical rib, while Stopford in 1918 found very marked similar changes in the distal arteries, following gunshot injuries to nerves much higher up in the limbs.

Some very interesting work has more recently been carried out by R. Cunliffe Shaw.[3] Very briefly these experiments, which were carried out in rabbits, consisted of the production of irritative lesions of the cardio-aortic nerves and subsequent investigation of the aorta. He first applied electrical stimulation externally from a 2-volt accumulator and induction coil along the vagal or sympathetic paths, the strength of the stimulus being just short of that which apparently produced pain. When the vagal path was irritated there was slowing of respiration and heart beat, while there was quickening of both these and prominence of the eyes when the sympathetic was irritated. The duration of the stimulus was ten to sixty minutes, with intervals of five or six days between the stimulations. Pathological changes occurred in every case, and included such lesions as dilatation of right ventricle, thickening of mitral valve, large and small nodular thickening of the aorta, and sclerosis of the cusps of the aortic valve.

The second method he used was to expose the nerves to the irritation of the air, and by the production of scar tissue around them. Twenty-two operations were performed on the vagal path, five on the sympathetic and five on the depressor. Unfortunately, all these nerves may have been affected in each case owing to the amount of scar tissue produced, and therefore it was impossible to say which fibres were the cause of the production of the lesions. Irritation of the vagi produced endarteritis of the aorta, especially of the first part in ten out of thirteen rabbits. Irritation of the sympathetic produced similar results in four out of five rabbits. Irritation of the depressor again produced similar results in two out of five, but two died at the operation.

"The pathological changes are constant ones, and progress through a definite sequence, the earliest consisting of a proliferation of the intima, in which both endothelium and areolar and elastic tissues participate. This is followed by hyperplasia of the elastic fibres in the media and early signs of degeneration in the smooth muscle cells. Fat is deposited in the intima, and the deep layers of this zone undergo necrosis, with the formation in advanced cases of atheromatous abscesses, sloughing of the inner layer of the intima, and the formation of ulcers."

### The Blood.

In many cases in which there is an over-action of the parasympathetic nervous system we find associated with it an eosinophilia. Asthma may be taken as a typical example. Why this should be I know of no answer. It is true that many of the causes of eosinophilia are associated with vagus irritation, *e.g.* worms in the intestine. But then again we meet with eosinophilia in such diseases as trichiniasis in muscles.

There is much to be learnt on the question, why different toxæmias should lead to different blood pictures, why in one complaint there should be leucocytosis, in others lymphocytosis, in others eosinophilia. Is there any evidence of nervous control of leucocytic activity by the autonomic nervous system? Mueller[4] has made the following observations :—

Injection of non-specific albumin preparations, of minute amounts of salt solution, of distilled water, or even of air intradermally, produces an immediate leucopenia in the whole of the peripheral circulation, which lasts from five to thirty minutes, the cells migrating to the splanchnic area. Fifty to seventy-five million cells migrate from one area to another.

About 0·2 cc. of a solution of albumin is required. If given subcutaneously, intramuscularly, or intravenously the reaction does not occur. Expressed in percentage, the decrease is not influenced by pre-existing conditions of the blood, *e.g.* in a normal person the drop is from 8,000 to 5,000, in conditions associated with leucocytosis (*e.g.* pneumonia) from 25,000 to 16,000, in leukæmia say from 150,000 to 80,000. A similar decrease is seen in conditions associated with leucopenia, *e.g.* pernicious anæmia 5,000 to 1,600.

The substances injected are unable by themselves to produce organic changes in the body. By blocking the nerves which carry the vaso-dilator fibres in the splanchnic area it is no longer possible to produce these changes. Atropin and adrenalin, if given subcutaneously in any part of the body, make it impossible to produce this reaction of influence on the leucocyte count by intradermal injection.

Adrenalin, as we know, stimulates the sympathetic system. Atropin paralyses the parasympathetic. Pilocarpin, on the other hand, which stimulates the parasympathetic, does not change the influence of the intradermal injections on the leucocytic reaction. In fact pilocarpin itself produces a drop, but a still further drop ensues when the intradermal injection is given.

The skin stimulation is supposed to be associated with a sudden change in the vascular system of the splanchnic area. If the nerves accompanying the large vessels going to a limb are severed, the intradermal injection in that limb no longer produces a general peripheral leucopenia. If, on the other hand, the intradermal injection is made into any other part of the body the diminution of leucocytes does occur in the general peripheral circulation, except in the extremity in which the nerves have been severed. These experiments show that the splanchnic area is not the only one involved. The part played in the extremity in which the nerves have been severed is not, therefore, merely a passive one. It was an active process which has been stopped by the severance of the nerves.

If a very diluted solution of adrenalin (1 in 100,000) is injected hypodermically a leucopenia is produced in the adjacent blood vessels, and curiously this only affects the polymorphonuclear leucocytes, the lymphocytes and other cells remaining as before.

The same author has made some most interesting observations on insulin shock in diabetic patients. Insulin shock shows itself by dizziness, weakness, trembling, profuse perspiration and collapse. As the blood sugar decreases so the leucocytes in the peripheral blood increase. In two cases which he observed the leucocytes reached 28,000 and 19,000. This increase from the normal occurred in fifteen minutes. In each case glucose was given (5 grammes in 100 cc. of water). In less than ten minutes the leucocytes had fallen to 7,000. He attributes the shock to parasympathetic overbalance, and says that there is a simultaneous sympathetic overbalance in the splanchnic area. He suggests that all these observations tend to show that the periphery, as represented by the skin, and the splanchnic area, as represented by the liver, are controlled by one centre, and that the sugar centre in the medulla may act in connection with this. A mutual control of the skin and splanchnic area may lead to completely new conceptions. "Where parasympathetic stimuli predominate in the vascular system," he writes, "there the leucocytes will accumulate, while an overbalance of sympathetic stimuli produces a decrease of leucocytes in this region. Both processes originate in the autonomic nervous system, the migration of leucocytes being but a sequel."

We must not, therefore, dogmatically assume that a blood film taken in the usual way from the peripheral circulation represents the state of the blood throughout the whole of the body.

REFERENCES.

[1] *Quarterly Journal Medicine*, 1925–26, vol. xix., p. 311.
[2] *Journal of Nervous and Mental Diseases*, 1914, vol. xli., p. 517.
[3] *Quarterly Journal Medicine*, 1925–26, vol. xix., p. 203.
[4] *Archives of Internat. Medicine*, 1926, vol. xxxvii., p. 268.

